# An Overt Chemical Protective Garment Reduces Thermal Strain Compared with a Covert Garment in Warm-Wet but Not Hot-Dry Environments

**DOI:** 10.3389/fphys.2017.00913

**Published:** 2017-11-09

**Authors:** Matthew J. Maley, Joseph T. Costello, David N. Borg, Aaron J. E. Bach, Andrew P. Hunt, Ian B. Stewart

**Affiliations:** ^1^Institute of Health and Biomedical Innovation, School of Exercise and Nutrition Sciences, Queensland University of Technology, Brisbane, QLD, Australia; ^2^Extreme Environments Laboratory, Department of Sport and Exercise Science, University of Portsmouth, Portsmouth, United Kingdom

**Keywords:** core temperature, clothing, heat stress, occupational work, personal protective equipment

## Abstract

**Objectives:** A commercial chemical, biological, radiological and nuclear (CBRN) protective covert garment has recently been developed with the aim of reducing thermal strain. A covert CBRN protective layer can be worn under other clothing, with equipment added for full chemical protection when needed. However, it is unknown whether the covert garment offers any alleviation to thermal strain during work compared with a traditional overt ensemble. Therefore, the aim of this study was to compare thermal strain and work tolerance times during work in an overt and covert ensemble offering the same level of CBRN protection.

**Methods**: Eleven male participants wore an overt (OVERT) or covert (COVERT) CBRN ensemble and walked (4 km·h^−1^, 1% grade) for a maximum of 120 min in either a wet bulb globe temperature [WBGT] of 21, 30, or 37°C (Neutral, WarmWet and HotDry, respectively). The trials were ceased if the participants' gastrointestinal temperature reached 39°C, heart rate reached 90% of maximum, walking time reached 120 min or due to self-termination.

**Results:** All participants completed 120 min of walking in Neutral. Work tolerance time was greater in OVERT compared with COVERT in WarmWet (*P* < 0.001, 116.5[9.9] vs. 88.9[12.2] min, respectively), though this order was reversed in HotDry (*P* = 0.003, 37.3[5.3] vs. 48.4[4.6] min, respectively). The rate of change in mean body temperature and mean skin temperature was greater in COVERT (0.025[0.004] and 0.045[0.010]°C·min^−1^, respectively) compared with OVERT (0.014[0.004] and 0.027[0.007]°C·min^−1^, respectively) in WarmWet (*P* < 0.001 and *P* = 0.028, respectively). However, the rate of change in mean body temperature and mean skin temperature was greater in OVERT (0.068[0.010] and 0.170[0.026]°C·min^−1^, respectively) compared with COVERT (0.059[0.004] and 0.120[0.017]°C·min^−1^, respectively) in HotDry (*P* = 0.002 and *P* < 0.001, respectively). Thermal sensation, thermal comfort, and ratings of perceived exertion did not differ between garments at trial cessation (*P* > 0.05).

**Conclusion:** Those dressed in OVERT experienced lower thermal strain and longer work tolerance times compared with COVERT in a warm-wet environment. However, COVERT may be an optimal choice in a hot-dry environment. These findings have practical implications for those making decisions on the choice of CBRN ensemble to be used during work.

## Introduction

Protection from chemical, biological, radiological and nuclear (CBRN) threats is a priority for hazmat teams and first responders deployed to potentially contaminated zones. Within these threat areas, an individual is unable to remove their CBRN protective ensemble and other personal protective equipment (PPE). As a consequence, an uncompensable environment is often experienced, with the internal danger of heat illness (Carter and Cammermeyer, 1985; Stewart et al., 2011) adding to the external danger (i.e., CBRN threat). Left unchecked, extreme deep body temperatures may result in organ failure and potentially death (Laing and Sleivert, 2002; Carter et al., 2005; Lucas et al., 2014).

The danger of heat illness arises as a result of the CBRN ensemble providing a physical barrier between the individual's skin surface and the environment, thus reducing heat loss from the body (McLellan et al., 2013). CBRN ensembles are often bulky, encapsulating and permit low water vapor permeability (Cheung et al., 2000). As an individual conducts work in these ensembles, air is trapped between the skin and ensemble, which warms, humidifies, and consequently impairs the avenues of heat loss, particularly evaporation (Nagata, 1978; Nunneley, 1989; Muza et al., 2001). The combination of these factors may result in a lethal combination of increased metabolic heat production (e.g., from added mass), impaired heat loss and an exacerbated rise in body temperature. Indeed, many studies have detailed the thermal strain during work in CBRN ensembles (Bishop et al., 1995; van den Heuvel et al., 2009; Blacker et al., 2013). Further, others have described the increased thermal strain and/or reduced work tolerance times in CBRN ensembles with warmer environmental temperatures in both field (Yokota et al., 2014) and laboratory trials (McLellan et al., 1993; Richmond et al., 2013; DenHartog et al., 2017).

CBRN ensembles are categorized on their level of protection and consequently differ on their level of permeability (Seed et al., 2008). The National Fire Protection Association (NFPA) 1991 and 1994 standards (2012; 2016) describe four classifications which vary in their level of encapsulation and CBRN protection. Unsurprisingly, previous research has demonstrated the correlation between the increased level of CBRN protection with reduced work tolerance times, as well as increased cardiovascular and thermal strain (McLellan et al., 1993; Montain et al., 1994; Yokota et al., 2014), highlighting the importance to optimize CBRN ensemble choice. Interestingly, CBRN ensemble comparisons within the same class (i.e., equivalent level of CBRN protection) may also result in differences in thermal strain (van den Heuvel et al., 2009) and work tolerance times (DenHartog et al., 2017).

A recent development has seen the commercial production of a covert CBRN garment that meets the NFPA 1994 (2012) standard if the hands and head are covered with appropriate additions. A covert CBRN protective layer can be worn under other clothing, with equipment added for full chemical protection when needed. The covert nature of the garment has enabled wearers to move freely through public areas without drawing attention or creating hysteria, often associated with the presence of military or emergency first responders in traditional overt CBRN ensembles.

Early studies showed that replacing an overt ensemble with CBRN protective combat clothing or a covert ensemble may extend work tolerance times in the heat (McLellan et al., 1992, 1994; Amos and Hansen, 1997). The improved tolerance to work may be a result of reduced trapped air layers, as well as improved water vapor permeability demonstrated with greater sweat evaporation compared with the respective overt ensemble. However, improved performance with a covert ensemble is not unanimous (Bomalaski et al., 1993). The covert ensembles utilized in these studies (McLellan et al., 1992, 1994; Bomalaski et al., 1993; Amos and Hansen, 1997) were prototypes and were compared to the highest level of protection. However, these ensembles would not meet the NFPA 1991 (2016) standard as the required breathing apparatus would not be encapsulated within the ensemble.

It is currently unknown whether commercially available covert ensembles are able to alleviate physiological strain during work compared with overt CBRN ensembles. Therefore, the aim of this study was to investigate the work tolerance times as well as physiological responses in varying environmental conditions between an overt and covert CBRN ensemble, both meeting the same NFPA 1994 (2012) class standard. It is hypothesized the covert ensemble will reduce thermal strain, resulting in longer work tolerance times compared with an overt CBRN ensemble.

## Methods

The present study was approved by the Queensland University of Technology's Human Research Ethics Committee (approval number: 1000001160) and complied with standards set in the Declaration of Helsinki. The participants were made aware of the purpose, procedures and risks of the study prior to giving their informed written consent. A total of 11 male participants volunteered. Their physical characteristics were as follows [mean (SD)]: 23 (3) years of age; height of 177.4 (5.5) cm; body mass of 77.4 (7.9) kg; maximal oxygen uptake (VO_2max_) of 56.9 (4.0) ml·kg^−1^·min^−1^. All participants were non-smokers and free from any vascular, blood, and respiratory conditions.

Eight participants completed both ensembles within an environment, however, these were not always the same eight between environments. Table 1 provides an overview of trials completed for each participant. Due to equipment failure in the warm-wet trial, only seven participants were included in the analysis of skin temperature and mean body temperature.

**Table 1:** Trial completion matrix.

**Environment**	**Neutral**		**WarmWet**		**HotDry**	
**Ensemble**	**OVERT**	**COVERT**	**OVERT**	**COVERT**	**OVERT**	**COVERT**
Participant 1	✓	✓	✓	✓	✓	✓
Participant 2	✓	✓			✓	✓
Participant 3	✓	✓	✓	✓	✓	✓
Participant 4	✓	✓			✓	✓
Participant 5	✓	✓	✓	✓	✓	✓
Participant 6	✓	✓			✓	✓
Participant 7	✓	✓	✓	✓	✓	✓
Participant 8	✓	✓	✓	✓	✓	✓
Participant 9			✓	✓		
Participant 10			✓	✓		
Participant 11			✓	✓		

Participants were instructed to refrain from alcohol, tobacco, caffeine and strenuous exercise, as well as to consume 45 mL of water per kg of body mass in the 24 h preceding each visit to the laboratory.

### Preliminary session

Participants' height and nude body mass were measured before performing a progressive incremental running test to exhaustion on a motorized treadmill to ascertain VO_2max_. Following a warm up period, participants were fitted with expired gas analysis equipment (Moxus, AEI Technologies, Pennsylvania, USA) and a heart rate (HR) monitor (Polar Team^2^, Kempele, Finland). The test started at a speed of ~9 km·h^−1^ and a 1% grade. On every minute, the speed was increased by 1 km·h^−1^ until a speed the participant could maintain for at least 2 min was established. Thereafter, the grade was increased by 1% every minute until volitional exhaustion was achieved. The variables used for determination of VO_2max_ followed the standard laboratory procedure and were as follows: plateau in VO_2_ (i.e., <150 mL·min^−1^ change with increase in workload); HR within 10 beats per minute of age predicted maximum (i.e., 220—age); respiratory exchange ratio >1.10; rating of perceived exertion ≥19. Where two criteria were met, two highest successive 15 s values for VO_2_ defined participants' VO_2max_.

Following the incremental test, participants were familiarized with the ensembles (detailed below) whilst walking on the treadmill at the speed to be utilized for the experimental sessions.

### Ensembles

During experimental trials, participants wore either an overt (OVERT) or covert (COVERT) ensemble, both adhering to the Class 3 NFPA 1994 (2012) standard. Specific details of ensembles:

1. OVERT: Emergency Response Suit (1.35 kg; Lion Apparel, Ohio, USA) consisting of a one-piece hooded jumpsuit, including inner gloves, booties and was worn with outer gloves and a respirator and filter (0.70 kg; Promask with a Pro2000 PF10 filter, Scott Safety, Lancashire, England). Combined ensemble mass of 2.05 kg.
2. COVERT: Chemical Protective Clothing System (1.40 kg; Lion Apparel, Ohio, USA) consisting of a jacket, trousers, a hood, booties and inner and outer gloves worn underneath a Nomex Flight Suit (0.85 kg; CWU 27/P, Propper, Missouri, USA). The Flight Suit is a one-piece garment which covers the torso and the full length of arms and legs. A respirator and filter were also worn (0.70 kg; as detailed above). Combined ensemble mass of 2.95 kg.

For the OVERT trial, participants wore a base ensemble which consisted of a t-shirt, shorts, socks and underwear. For the COVERT trial, the base ensemble was the same as detailed above except no t-shirt was worn. Athletic shoes with a soft rubber sole were also worn during testing. These base ensemble requirements were standardized in accordance with the American Society for Testing and Materials guidelines (2007).

### Experimental sessions

The experimental sessions involved walking for up to 120 min on a motorized treadmill at a speed of 4 km·h^−1^ with a 1% gradient. Presentation of ensembles and environmental conditions were randomly assigned. The experimental sessions were conducted in a climate controlled chamber maintained at a wet bulb globe temperature (WBGT) of 21°C (Neutral), 30°C (WarmWet), or 37°C (HotDry) obtained by the following dry bulb temperatures and relative humidities (rh): 24°C, 50%; 32°C, 60%; and 48°C, 20%, respectively. A wind speed equivalent to ~4.7 km·h^−1^ and a radiant heat load, from radiant heaters positioned ~1.3 m above and either side of the participant, were incorporated throughout all trials. WBGT (Quest Temp, Airmet, Australia) and wind speed (Kestrel 4000, KestrelMeters, Minnesota, USA) were monitored at the level of the participants' waist.

Standard termination criteria were applied during each trial in accordance with the American Society for Testing and Materials guidelines (2007): (1) deep body temperature >39.0°C; (2) 120 min of exercise; (3) HR ≥ 90% of maximum; or (4) fatigue or nausea (self-termination). Following the attainment of one of the termination criteria, the participant exited the environmental chamber and removed the ensemble.

### Measurements

Pre-trial hydration status was confirmed as urine specific gravity (USG; PAL 10 s, ATAGO, Tokyo, Japan) of < 1.020 (Armstrong, 2005). If participants did not meet this guideline they were given an additional 500 mL of tap water, which was consumed 30 min prior to the commencement of the trial. Nude body mass was measured prior to exercise and on completion of the trial, following complete towel drying to remove surface sweat. Deep body temperature was estimated using a gastrointestinal pill (T_gi_) ingested 6 h prior to the experimental trials and recorded at 30 s intervals (CorTemp, HQ Inc., Palmetto, FL, USA). All ingestible pills were calibrated and raw data corrected as previously described (Hunt and Stewart, 2008; Hunt et al., 2017). Mean skin temperature (${\bar{\text{T}}}_{\text{msk}}$) was estimated using iButton thermocrons (DS1922L-F50 iButtons, Maxim Integrated, California, USA) attached to four locations (i.e., back of neck, inferior border of right scapula, dorsal left, hand and proximal third of right tibia) (ISO 9886) (2004) and recorded at 30 s intervals. HR was measured using a chest strap and monitor (Polar Team^2^, Kempele, Finland) and recorded at 30 s intervals.

Thermal sensation was assessed using a modified scale (Gagge et al., 1967), where 1 had the anchor of “extremely cold” and 13 “unbearably hot.” Similarly, thermal comfort was assessed using a modified scale (Gagge et al., 1967), where 1 had the anchor of “comfortable” and 5 “extremely uncomfortable.” Rating of perceived exertion was assessed using the Borg 15-point scale (Borg, 1982), where 6 had the anchor of “very, very light” and 20 “maximal exertion.” All perceptual data were recorded every 15 min, with participant's final values recorded at the cessation of exercise.

### Calculations

Mean skin temperature (${\bar{\text{T}}}_{\text{msk}}$) was calculated as [ISO 9886 (2004)]:

$$\begin{array}{l} {\bar{\text{T}}}_{\text{msk}}=0.28{\text{T}}_{\text{neck}}\;+\;0.28{\text{T}}_{\text{scapula}}\;+\;0.16{\text{T}}_{\text{hand}}\;+\;0.28{\text{T}}_{\text{shin}} \end{array}$$

Mean body temperature (${\bar{\text{T}}}_{\text{b}}$) was calculated as Hardy and Du Bois (1938b):

$$\begin{array}{l} {\bar{\text{T}}}_{\text{b}}=0.8{\text{T}}_{\text{gi}}\;+\;0.2{\bar{\text{T}}}_{\text{msk}} \end{array}$$

### Statistical analyses

Statistical analyses were conducted using SPSS version 23 for Windows (IBM Corporation, New York, USA). An α of 0.05 was used to determine statistical significance. Data were assessed for normality with a Shapiro-Wilk test and visual inspection of data (e.g., boxplots). A univariate general linear model was utilized with model factors comprising “participant” as a random factor and “ensemble” and “environment” as fixed factors. Where a significant main effect for environment was observed, pairwise comparisons were used to investigate differences in the dependent variable, with Bonferroni adjustments applied for multiple comparisons. Where a significant interaction was observed, pairwise comparisons were used to investigate the within-group differences in the dependent variable.

## Results

Participants commenced exercise euhydrated, with all physiological data similar between trials (Table 2, all *P* > 0.05).

**Table 2:** Mean (SD) baseline physiological and hydration indices within each environment and ensemble.

**Environment**	**Neutral**		**WarmWet**		**HotDry**	
**Ensemble**	**OVERT**	**COVERT**	**OVERT**	**COVERT**	**OVERT**	**COVERT**
HR (bpm)	81 (11)	76 (8)	84 (10)	78 (7)	84 (8)	80 (6)
T_gi_ (°C)	37.1 (0.3)	37.1 (0.2)	37.1 (0.3)	37.0 (0.2)	37.3 (0.3)	37.0 (0.3)
${\bar{\text{T}}}_{\text{msk}}$ (°C)	33.2 (0.6)	33.2 (0.5)	33.5 (0.9)	33.5 (0.7)	33.3 (0.8)	33.2 (0.5)
Body mass (kg)	79.9 (7.3)	80.5 (7.5)	78.5 (8.3)	77.7 (8.5)	80.0 (7.7)	80.4 (7.5)
USG	1.012 (0.005)	1.012 (0.007)	1.013 (0.007)	1.009 (0.006)	1.014 (0.005)	1.014 (0.005)

*HR, heart rate; T_gi_, gastrointestinal temperature; ${\bar{\text{T}}}_{\text{msk}}$, mean skin temperature; USG, urine specific gravity*.

### Work tolerance times and termination criteria

**Figure 1:**
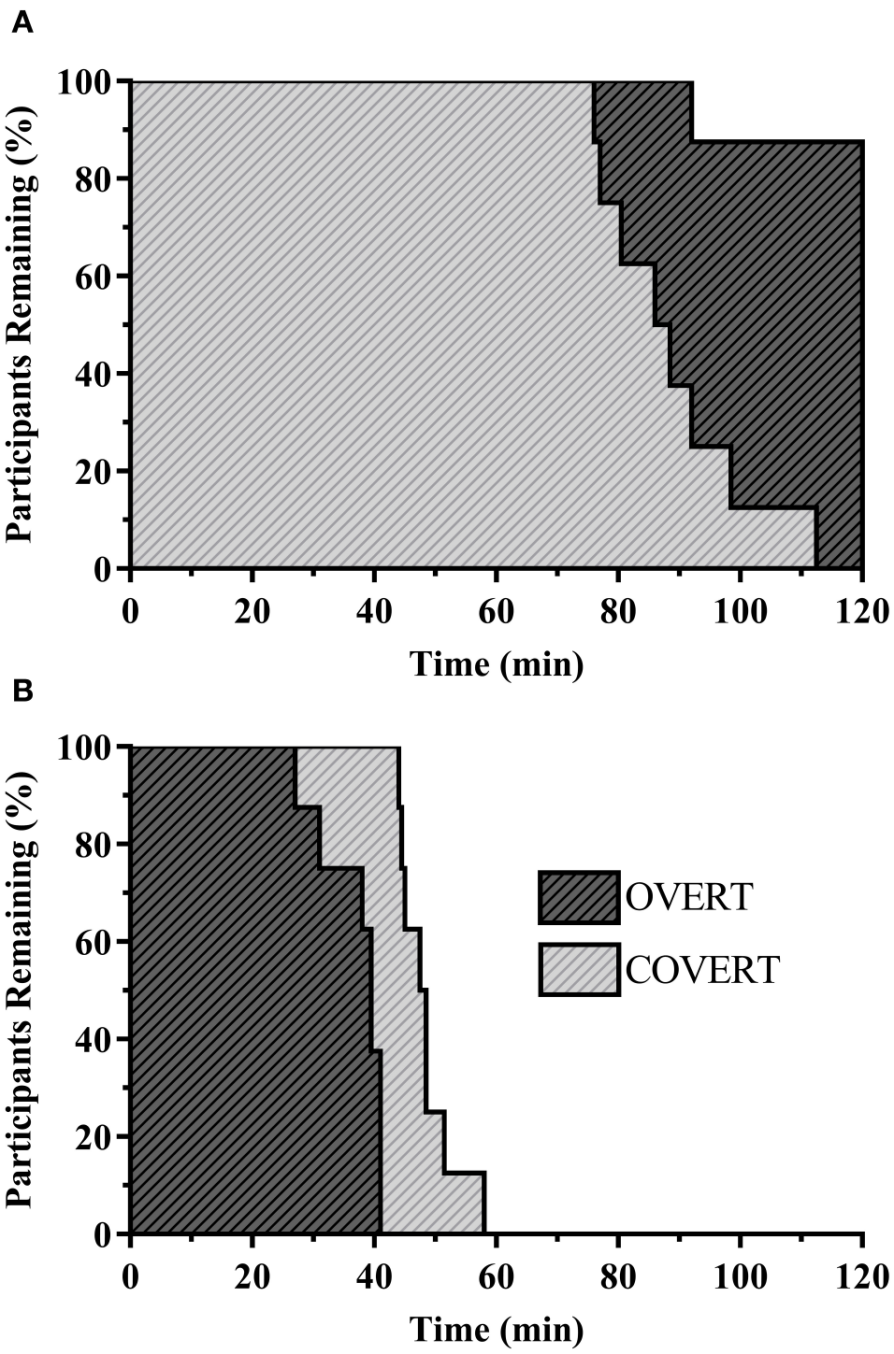
Individual work tolerance time for participants wearing CBRN ensembles in WarmWet **(A)** and HotDry **(B)**.

An overview of work tolerance times for WarmWet and HotDry is shown in Figures 1A,B respectively. There was a significant main effect for environment (Table 3, *P* < 0.001) and ensemble (*P* = 0.012), as well as an interaction of both factors (*P* < 0.001). Pairwise comparisons revealed participants exercised for longer in Neutral vs. WarmWet (*P* < 0.001), with participants in the latter environment exercising for longer than HotDry (*P* < 0.001). When considering the interaction of both factors, pairwise comparisons revealed in WarmWet participants exercised for longer in OVERT vs. COVERT (Table 3, *P* < 0.001). However, in HotDry, participants exercised for longer in COVERT vs. OVERT (*P* = 0.003).

**Table 3:** Mean (SD) work tolerance times and the number of participants meeting each termination criteria within each environment and ensemble.

**Environment**	**Neutral**		**WarmWet**			**HotDry**		
**Ensemble**	**OVERT**	**COVERT**	**OVERT**		**COVERT**	**OVERT**		**COVERT**
Work tolerance time (minutes)^†^^‡^^§^	120.0 (0.0)	120.0 (0.0)	116.5 (9.9)	^*^	88.9 (12.2)	37.3 (5.3)	^*^	48.4 (4.6)
HR (≥90% max)	–	–	–		–	2		–
T_gi_ (>39°C)	–	–	1		5	6		8
Self-termination	–	–	–		3	–		–
Duration (120 min)	8	8	7		–	–		–

HR, heart rate; T_gi_, gastrointestinal temperature.

† main effect for environment,

‡ main effect for ensemble,

§ significant interaction,

* *significant difference between ensembles within environment (P < 0.05)*.

### Physiological strain at the cessation of trial

At the cessation of trial, there was a main effect for environment for all physiological variables measured (Table 4, all *P* < 0.001), whilst a significant main effect for ensemble was observed for HR (*P* = 0.018), T_gi_ (*P* = 0.022), ${\bar{\text{T}}}_{\text{msk}}$ rate of change (*P* = 0.021) and ${\bar{\text{T}}}_{\text{b}}$ (*P* = 0.014). There was a significant interaction for T_gi_ rate of change (*P* = 0.003), ${\bar{\text{T}}}_{\text{msk}}$ (*P* < 0.001), ${\bar{\text{T}}}_{\text{msk}}$ rate of change (*P* < 0.001), T_gi_ to ${\bar{\text{T}}}_{\text{msk}}$ gradient (*P* < 0.001), ${\bar{\text{T}}}_{\text{b}}$ (*P* = 0.017), ${\bar{\text{T}}}_{\text{b}}$ rate of change (*P* < 0.001), body mass loss (*P* = 0.016) and sweat rate (*P* = 0.019).

**Table 4:** Mean (SD) physiological strain at the cessation of trial within each environment and ensemble.

**Environment**	**Neutral**		**WarmWet**			**HotDry**		
**Ensemble**	**OVERT**	**COVERT**	**OVERT**		**COVERT**	**OVERT**		**COVERT**
**HR (bpm)**^†^^‡^	100 (7)	110 (10)	145 (19)		164 (8)	161 (16)		161 (17)
**T**_gi_ **(**°**C)**^†^^‡^	37.6 (0.2)	37.7 (0.3)	38.5 (0.5)		38.9 (0.2)	38.9 (0.2)		39.1 (0.0)
**T**_gi_ **(**°**C**·**min**^−1^**)**^†^^§^	0.004 (0.002)	0.005 (0.003)	0.012 (0.006)	^*^	0.021 (0.004)	0.045 (0.009)		0.042 (0.003)
$\bar{\text{T}}$_msk_ **(**°**C)**^†^^§^	34.7 (0.4)	35.0 (0.3)	36.8 (0.4)	^*^	37.5 (0.4)	39.5 (0.2)	^*^	39.0 (0.3)
$\bar{\text{T}}$_msk_ **(**°**C**·**min**^−1^**)**^†^^‡^^§^	0.013 (0.007)	0.015 (0.005)	0.027 (0.007)	^*^	0.045 (0.010)	0.170 (0.026)	^*^	0.120 (0.017)
**T**_gi_ **to** $\bar{\text{T}}$_msk_ **gradient**^†^^§^	2.9 (0.4)	2.7 (0.4)	1.6 (0.4)		1.4 (0.4)	−0.6 (0.2)	^*^	0.1 (0.3)
$\bar{\text{T}}$_b_ **(**°**C)**^†^^‡^^§^	37.1 (0.2)	37.1 (0.3)	38.1 (0.4)	^*^	38.6 (0.2)	39.1 (0.2)		39.0 (0.1)
$\bar{\text{T}}$_b_ **(**°**C**·**min**^−1^**)**^†^^§^	0.006 (0.003)	0.007 (0.003)	0.014 (0.004)	^*^	0.025 (0.004)	0.068 (0.010)	^*^	0.059 (0.004)
**Body mass loss (%)**^†^^§^	1.0 (0.2)	1.4 (0.4)	2.7 (0.6)	^*^	2.3 (0.3)	1.4 (0.3)		1.5 (0.2)
**Sweat rate (% body mass loss**·**h**^−1^**)**^†^^§^	0.5 (0.1)	0.7 (0.2)	1.4 (0.3)		1.6 (0.2)	2.2 (0.4)	^*^	1.9 (0.3)

HR, heart rate; T_gi_, gastrointestinal temperature; ${\bar{\text{T}}}_{\text{msk}}$, mean skin temperature; ${\bar{T}}_{\text{b}}$, mean body temperature.

† main effect for environment,

‡ main effect for ensemble,

§ significant interaction,

* *Significant difference between ensembles within environment (P < 0.05)*.

In WarmWet, COVERT, compared with OVERT, had a higher ${\bar{\text{T}}}_{\text{msk}}$ (*P* < 0.001), ${\bar{\text{T}}}_{\text{b}}$ (*P* < 0.001), lower body mass loss (*P* = 0.039), a faster rate of change in T_gi_ (*P* < 0.001), ${\bar{\text{T}}}_{\text{msk}}$ (*P* = 0.028) and ${\bar{\text{T}}}_{\text{b}}$ (*P* < 0.001). In HotDry, OVERT, compared with COVERT, had a higher ${\bar{\text{T}}}_{\text{msk}}$ (*P* = 0.001), a negative T_gi_ to ${\bar{\text{T}}}_{\text{msk}}$ gradient (*P* < 0.001), faster rate of change in ${\bar{\text{T}}}_{\text{msk}}$ (*P* < 0.001) and ${\bar{\text{T}}}_{\text{b}}$ (*P* = 0.002), as well as a greater sweat rate (*P* = 0.032).

### Perceptual data at the cessation of trial

**Figure 2:**
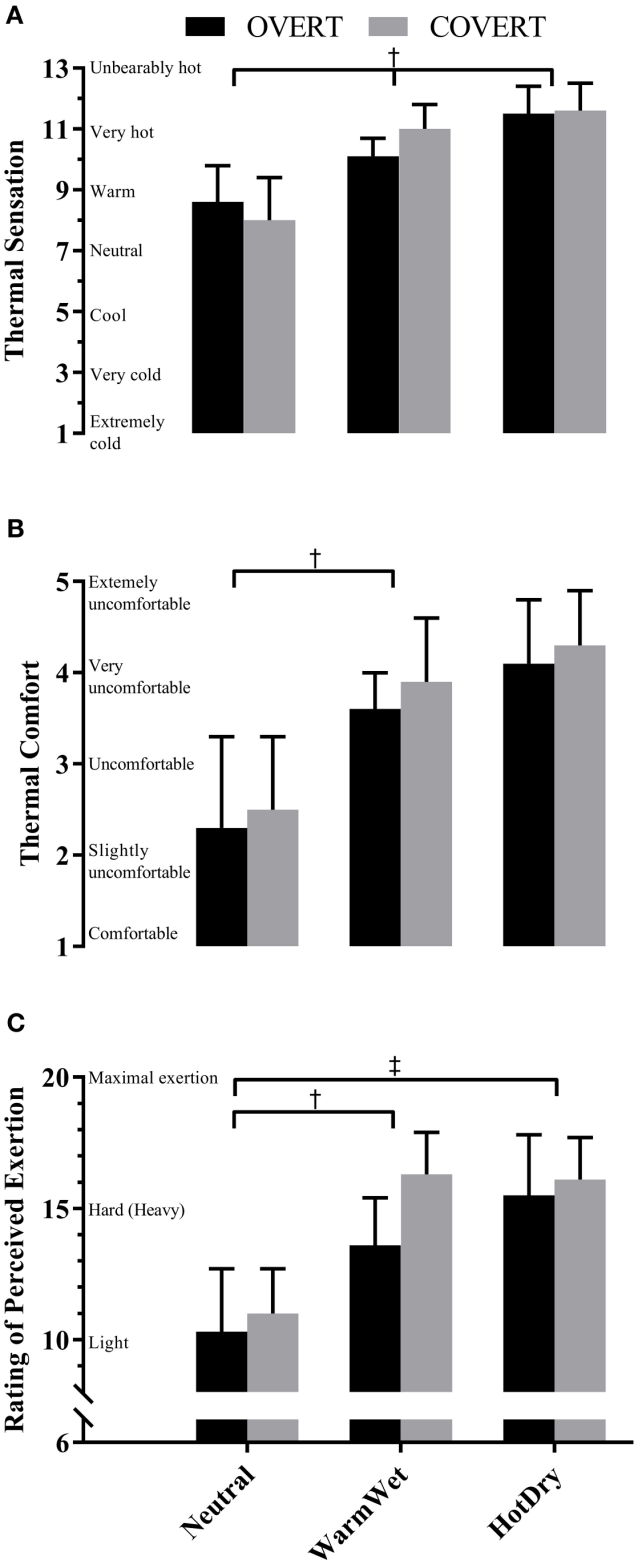
Mean (SD) thermal sensation **(A)**, thermal comfort **(B)** and rating of perceived exertion **(C)** in each CBRN ensemble and environment. ^†^main effect for environment, ^‡^main effect for ensemble (*P* < 0.05).

At the cessation of the trial, there was a main effect for environment for thermal sensation, thermal comfort and RPE (Figure 2, all *P* < 0.001). Pairwise comparisons for thermal sensation revealed participants felt warmer as WBGT increased (Neutral < WarmWet < HotDry, all *P* < 0.05). Pairwise comparisons for thermal comfort and RPE revealed participants felt more uncomfortable and reported a greater perceived exertion in WarmWet vs. Neutral (*P* < 0.001); however, there was no difference between WarmWet and HotDry (*P* > 0.05). A main effect for ensemble was observed for RPE only, with participants reporting a greater perceived exertion in COVERT vs. OVERT (*P* = 0.020). No significant interactions were observed for any perceptual variables (*P* > 0.05).

## Discussion

The novel finding from this study was that in a warm-wet environment work tolerance times were longer in OVERT compared with COVERT, whilst in a hot-dry environment the order was reversed. As such, work tolerance times appear to be both environment and ensemble configuration dependent. Contrary to our hypothesis these data suggest that, depending on environmental conditions, COVERT may not be an optimal alternative to OVERT.

Individuals in Neutral were able to complete the duration of the test in either ensemble (Table 3). In WarmWet, individuals worked for 31% (~28 min) longer in OVERT vs. COVERT. While participants exercised for 30% longer in COVERT compared with OVERT in HotDry, the improvement in absolute work tolerance time was 11 min. Despite this being lower than the improvement observed in WarmWet, the increase in work tolerance time is of practical benefit to workers dressed in CBRN ensembles. Given that the same workload was employed between trials, the reason for the difference in times and reversal of order may be due to differences in ensemble properties.

An individual's thermoregulation whilst dressed in these CBRN ensembles may be affected by a wide range of clothing factors including insulation, air and water vapor permeability as well as skin coverage, layering and fit of the ensemble (Havenith, 1999). Multiple clothing layers trap air creating a microenvironment, subsequently decreasing heat loss through layers (Sullivan and Mekjavić, 1992; Havenith, 1999). While the outer layer of both ensembles covered the surface area of the body, the CBRN protective undergarment in the COVERT trial also covered most of the individual's body, leaving only the hands, feet and head free from coverage. A greater coverage of the body increases total thermal resistance (insulation) (Parsons, 2003), resulting in reduced dry heat exchange between the skin and environment leading to greater ${\bar{\text{T}}}_{\text{msk}}$.

When compared with OVERT, there was a faster rate of change in ${\bar{\text{T}}}_{\text{msk}}$ in COVERT during WarmWet (Table 4) with the order reversed in HotDry. With dry heat exchange already compromised in COVERT (due to double layering) it is paradoxical to observe a slower rate of change in ${\bar{\text{T}}}_{\text{msk}}$ vs. OVERT in HotDry. Since ambient air temperature is warmer than skin temperature in HotDry, slowing the rate of change in ${\bar{\text{T}}}_{\text{msk}}$ would require manipulation of sweat evaporation as this is the primary avenue of heat loss under this circumstance (Hardy and Du Bois, 1938a). However, we are unable to speculate further as there was no measure of sweat efficiency nor any testing made on a sweating thermal manikin in the present study.

Irrespective of the ensemble, work tolerance times and physiological strain were significantly influenced by the environment in a dose-dependent manner (Table 3). The literature has consistently demonstrated reduced work tolerance times with increasing ambient temperatures and/or humidity in both PPE and/or CBRN protective ensembles (McLellan et al., 1996, 2013; Stewart et al., 2014; Costello et al., 2015a,b; DenHartog et al., 2017). Compared with COVERT in WarmWet, McLellan et al. (1993) reported similar work tolerance times (83 min, in 30°C, 50% rh) which included similar body surface area coverage to the present study. However, it is difficult to directly compare the present study to previous investigations given the wide range of ensembles and environmental conditions tested.

Previous studies utilizing covert ensembles during continuous walking have reported both longer (McLellan et al., 1994) and shorter (Bomalaski et al., 1993) work tolerance times compared to the present study. Both studies utilized similar walking speed to the present study, whilst the participants in the Bomalaski et al. (1993) study were older (mean [SD]: 35 [5] years of age) than the participants in the McLellan et al. (1994) and present study, which may have contributed to the shorter work tolerance time. It is not clear why the participants in the McLellan et al. (1994) study walked on average 39 min longer than the present study. Dry bulb temperature in the present study was 8°C greater than utilized by McLellan et al. (1994) but both had similar water vapor pressures (i.e., 2.2 kPa) which would primarily govern work tolerance times at this metabolic rate (McLellan et al., 2013) as dry heat loss in both environments were negligible (Hardy and Du Bois, 1938a). Though untested, it is speculated that ensemble properties, such as air permeability (Havenith et al., 2011), may differ between the prototype used by McLellan et al. (1994) and COVERT used in the present study.

An advantage of the covert CBRN protective layer utilized in the present study is that it is able to be worn discreetly, with additional PPE added to meet Class 3 NFPA 1994 (2012) standard. However, the authors are aware the present study did not include a period of time (resting and during walking) which compared the covert CBRN protective layer worn under the Nomex flight suit vs. “normal” (e.g., cotton) clothing alone in each environment. This may be important as the covert CBRN protective layer will increase thermal and vapor resistance relative to “normal” clothing alone, and may inadvertently predispose individuals to greater initial deep body temperature at the start of work (Mclellan, 2008).

While COVERT may outperform OVERT in a hot-dry environment, presumably because of an increased efficiency to evaporate sweat, this advantage may not translate to all individuals. For example, the participants in the present study represent individuals who are young, fit and healthy and thus experience greater sweat rates than older individuals (Smith et al., 2013; Stapleton et al., 2014), who may not be able to take full advantage of the reduced vapor resistance offered by COVERT. Future research should include an age comparison using the present study's ensembles and environmental conditions to ensure any recommendations on ensemble choice are tailored for a wide range of individuals.

While the present study did not utilize a repeated measures design, analysis of work tolerance times of the five participants who completed all three environments and both ensembles (see Table 1) revealed the same outcome as the present study. That is, all participants completed 120 min walking in Neutral in both ensembles, whilst in WarmWet work tolerance times were longer in OVERT vs. COVERT, though this order was reversed in HotDry.

Based on the observations in the present study it is concluded that OVERT may be the optimal choice when performing work in a warm-wet environment, whereas COVERT would be recommended in a hot-dry environment. These findings have practical implications for those making decisions on the choice of CBRN ensemble to be used during work.

## Author contributions

IS and JC designed the research protocol. DB and AB collected the data. All authors interpreted results of experiments. MM drafted the manuscript. All authors approved the submitted manuscript and agreed to be accountable for all aspects of the work.

### Conflict of interest statement

The authors declare that the research was conducted in the absence of any commercial or financial relationships that could be construed as a potential conflict of interest.

## References

[B1] International Organisation for Standardisation 9886 (2004). Ergonomics — Evaluation of Thermal Strain by Physiological Measurements. Geneva: International Organisation for Standardisation.

[B2] Amos D. Hansen R. (1997). The physiological strain induced by a new low burden chemical protective ensemble. Aviat. Space Environ. Med. 68, 126–131. 9125088

[B3] Armstrong L. E. (2005). Hydration assessment techniques. Nutr. Rev. 63, S40–S54. 10.1301/nr.2005.jun.S40 16028571

[B4] American Society for Testing and Materials guidelines Standard F,668-07 (2007). Determining the Physiological Responses of the Wearer to Protective Clothing Ensembles. West Conshohocken, PA: ASTM International.

[B5] Bishop P. Ray P. Reneau P. (1995). A review of the ergonomics of work in the US military chemical protective clothing. Int. J. Ind. Ergon. 15, 271–283. 10.1016/0169-8141(94)00041-Z

[B6] Blacker S. D. Carter J. M. Wilkinson D. M. Richmond V. L. Rayson M. P. Peattie M. (2013). Physiological responses of police officers during job simulations wearing chemical, biological, radiological and nuclear personal protective equipment. Ergonomics 56, 137–147. 10.1080/00140139.2012.734335 23140326

[B7] Bomalaski S. H. Hengst R. Constable S. H. (1993). Thermal Stress in Seven Types of Chemical Defense Ensembles during Moderate Exercise in Hot Environments. US Air Force Armstrong Laboratories.

[B8] Borg G. A. (1982). Psychophysical bases of perceived exertion. Med. Sci. Sports Exerc. 14, 377–381. 10.1249/00005768-198205000-00012 7154893

[B9] Carter B. J. Cammermeyer M. (1985). Emergence of real casualties during simulated chemical warfare training under high heat conditions. Mil. Med. 150, 657–663. 3935970

[B10] Carter R. Cheuvront S. N. Williams J. O. Kolka M. A. Stephenson L. A. Sawka M. N.. (2005). Epidemiology of hospitalizations and deaths from heat illness in soldiers. Med. Sci. Sports Exerc. 37, 1338–1344. 10.1249/01.mss.0000174895.19639.ed 16118581

[B11] Cheung S. S. McLellan T. M. Tenaglia S. (2000). The thermophysiology of uncompensable heat stress. Physiological manipulations and individual characteristics. Sport Med. 29, 329–359. 10.2165/00007256-200029050-00004 10840867

[B12] Costello J. T. Stewart K. L. Stewart I. B. (2015a). Inside the “Hurt Locker”: the combined effects of explosive ordnance disposal and chemical protective clothing on physiological tolerance time in extreme environments. Ann. Occup. Hyg. 59, 922–931. 10.1093/annhyg/mev029 25878167

[B13] Costello J. T. Stewart K. L. Stewart I. B. (2015b). The effects of metabolic work rate and ambient environment on physiological tolerance times while wearing explosive and chemical personal protective equipment. Biomed. Res. Int. 2015:857536. 10.1155/2015/857536 25866818 PMC4383354

[B14] DenHartog E. A. Rubenstein C. D. Deaton A. S. Bogerd C. P. (2017). Variability in heat strain in fully encapsulated impermeable suits in different climates and at different work loads. Ann. Work Expo. Health 61, 248–259. 10.1093/annweh/wxw019 28395350

[B15] Gagge A. P. Stolwijk J. A. J. Hardy J. D. (1967). Comfort and thermal sensations and associated physiological responses at various ambient temperatures. Environ. Res. 1, 1–20. 10.1016/0013-9351(67)90002-3 5614624

[B16] Hardy J. D. Du Bois E. F. (1938a). Basal metabolism, radiation, convection and vaporization at temperatures of 22 to 35°C. J. Nutr. 15, 477–497.

[B17] Hardy J. D. Du Bois E. F. (1938b). The technic of measuring radiation and convection. J. Nutr. 15, 461–475.

[B18] Havenith G. (1999). Heat balance when wearing protective clothing. Ann. Occup. Hyg. 43, 289–296. 10.1016/S0003-4878(99)00051-4 10481628

[B19] Havenith G. den Hartog E. Martini S. (2011). Heat stress in chemical protective clothing: porosity and vapour resistance. Ergonomics 54, 497–507. 10.1080/00140139.2011.558638 21547794

[B20] Hunt A. P. Bach A. J. E. Borg D. N. Costello J. T. Stewart I. B. (2017). The systematic bias of ingestible core temperature sensors requires a correction by linear regression. Front. Physiol. 8:260. 10.3389/fphys.2017.00260 28496414 PMC5406512

[B21] Hunt A. P. Stewart I. B. (2008). Calibration of an ingestible temperature sensor. Physiol. Meas. 29, 71–78. 10.1088/0967-3334/29/11/N01 18843163

[B22] Laing R. M. Sleivert G. G. (2002). Clothing, textiles, and human performance. Text. Prog. 32, 1–122. 10.1080/00405160208688955

[B23] Lucas R. A. I. Epstein Y. Kjellstrom T. (2014). Excessive occupational heat exposure: a significant ergonomic challenge and health risk for current and future workers. Extrem. Physiol. Med. 3:14. 10.1186/2046-7648-3-14 25057350 PMC4107471

[B24] Mclellan T. M. (2008). Chemical-biological protective clothing: effects of design and initial state on physiological strain. Aviat. Space Environ. Med. 79, 500–508. 10.3357/ASEM.2211.2008 18500047

[B25] McLellan T. M. Bell D. G. Dix J. K. (1994). Heat strain with combat clothing worn over a chemical defense (CD) vapor protective layer. Aviat. Space Environ. Med. 65, 757–763. 7980339

[B26] McLellan T. M. Daanen H. A. M. Cheung S. S. (2013). Encapsulated environment. Compr. Physiol. 3, 1363–1391. 10.1002/cphy.c130002 23897690

[B27] McLellan T. M. Jacobs I. Bain J. B. (1993). Influence of temperature and metabolic rate on work performance with Canadian Forces NBC clothing. Aviat. Space Environ. Med. 64, 587–594. 8357310

[B28] McLellan T. M. Meunier P. Livingstone S. (1992). Influence of a new vapor protective clothing layer on physical work tolerance times at 40°C. Aviat. Space Environ. Med. 63, 107–113. 1546937

[B29] McLellan T. M. Pope J. I. Cain J. B. Cheung S. S. (1996). Effects of metabolic rate and ambient vapour pressure on heat strain in protective clothing. Eur. J. Appl. Physiol. Occup. Physiol. 74, 518–527. 10.1007/BF02376767 8971493

[B30] Montain S. J. Sawka M. N. Cadarette B. S. Quigley M. D. McKay J. M. (1994). Physiological tolerance to uncompensable heat stress: effects of exercise intensity, protective clothing, and climate. J. Appl. Physiol. 77, 216–222. 10.21236/ADA283851 7961236

[B31] Muza S. R. Banderet L. E. Cadarette B. (2001). Protective uniforms for nuclear, biological, and chemical warfare: metabolic, thermal, respiratory, and psychological issues, in Medical Aspects of Harsh Environments. Vol. 2, eds Pandolf K. B. Burr R. E. (Washington, D.C.: TMM Publications), 1084–1127.

[B32] Nagata H. (1978). Evaporative heat loss and clothing. J. Hum. Ergol. (Tokyo) 7, 169–175. 756450

[B33] NFPA 1991 (2016). Standard on Vapor-Protective Ensembles for Hazardous Materials Emergencies. National Fire Protection Association.

[B34] NFPA 1994 (2012). Standard on Protective Ensembles for First Responders to CBRN Terrorism Incidents. National Fire Protection Association.

[B35] Nunneley S. A. (1989). Heat stress in protective clothing. Interactions among physical and physiological factors. Scand. J. Work Environ. Health 15, 52–57. 2692140

[B36] Parsons K. (2003). Human Thermal Environments, 2nd Edn. London: Taylor and Francis.

[B37] Richmond V. L. Wilkinson D. M. Blacker S. D. Horner F. E. Carter J. Havenith G.. (2013). Insulated skin temperature as a measure of core body temperature for individuals wearing CBRN protective clothing. Physiol. Meas. 34, 1531–1543. 10.1088/0967-3334/34/11/1531 24149937

[B38] Seed M. Anand S. Kandola B. Fulford R. (2008). Chemical, biological, radiological and nuclear protection. Tech. Text. Int. 17, 39–46. Available online at: http://www.technical-textiles.net/news/chemical-biological-radiological-and-nuclear-protection

[B39] Smith C. J. Alexander L. M. Kenney W. L. (2013). Nonuniform, age-related decrements in regional sweating and skin blood flow. Am. J. Physiol. Regul. Integr. Comp. Physiol. 305, R877–R885. 10.1152/ajpregu.00290.2013 23926135 PMC3798768

[B40] Stapleton J. M. Fujii N. Carter M. Kenny G. P. (2014). Diminished nitric oxide-dependent sweating in older males during intermittent exercise in the heat. Exp. Physiol. 99, 921–932. 10.1113/expphysiol.2013.077644 24706193

[B41] Stewart I. B. Rojek A. M. Hunt A. R. (2011). Heat strain during explosive ordnance disposal. Mil. Med. 176, 959–963. 10.7205/MILMED-D-11-00052 21882791

[B42] Stewart I. B. Stewart K. L. Worringham C. J. Costello J. T. (2014). Physiological tolerance times while wearing explosive ordnance disposal protective clothing in simulated environmental extremes. PLoS ONE 9:e83740. 10.1371/journal.pone.0083740 24586228 PMC3931617

[B43] Sullivan P. J. Mekjavić I. B. (1992). Temperature and humidity within the clothing microenvironment: determinates of heat strain. Aviat. Space Environ. Med. 63, 186–192. 1567319

[B44] van den Heuvel A. Caldwell J. Patterson M. Taylor N. A. S. (2009). Physiological impact of first-responder chemical, biological and radiological protective ensembles, in Thirteenth International Conference on Environmental Ergonomics (Boston, MA), 39–43.

[B45] Yokota M. Karis A. J. Tharion W. J. (2014). Thermal-work strain in law enforcement personnel during chemical, biological, radiological, and nuclear (CBRN) training. Int. J. Occup. Environ. Health 20, 126–133. 10.1179/2049396714Y.0000000056 24999847 PMC4060587

